# African-American and Caucasian participation in postmortem human brain donation for neuropsychiatric research

**DOI:** 10.1371/journal.pone.0222565

**Published:** 2019-10-23

**Authors:** Amy Deep-Soboslay, Michelle I. Mighdoll, Andrew E. Jaffe, Stephen B. Thomas, Mary M. Herman, Jonathan Sirovatka, Jewell P. King, David R. Fowler, Dawn Zulauf, Constance DiAngelo, Thomas M. Hyde, Joel E. Kleinman

**Affiliations:** 1 Lieber Institute for Brain Development, Baltimore, Maryland, United States of America; 2 Johns Hopkins University, School of Public Health, Department of Mental Health, Baltimore, Maryland, United States of America; 3 University of Maryland, School of Public Health, Department of Health Policy & Management, Center for Health Equity, College Park, Maryland, United States of America; 4 Human Brain Collection Core, NIMH, NIH, Bethesda, Maryland, United States of America; 5 Office of the Chief Medical Examiner of the State of Maryland, Baltimore, Maryland, United States of America; 6 Office of the Chief Medical Examiner of Virginia, Northern District, Manassas, Virginia, United States of America; 7 Johns Hopkins University, School of Medicine, Department of Psychiatry and Behavioral Sciences, Baltimore, Maryland, United States of America; 8 Johns Hopkins University, School of Medicine, Department of Neurology, Baltimore, Maryland, United States of America; West Virginia University, UNITED STATES

## Abstract

Increased African-American research participation is critical to the applicability and generalizability of biomedical research, as population diversity continues to increase both domestically and abroad. Yet numerous studies document historical origins of mistrust, as well as other barriers that may contribute to resistance in the African-American community towards participation in biomedical research. However, a growing body of more recent scientific evidence suggests that African-Americans value research and are willing to participate when asked. In the present study, we set out to determine factors associated with research participation of African-American families in postmortem human brain tissue donation for neuropsychiatric disorders as compared with Caucasian families, from same-day medical examiner autopsy referrals. We retrospectively reviewed brain donation rates, as well as demographic and clinical factors associated with donation in 1,421 consecutive referrals to three medical examiner’s offices from 2010–2015. Overall, 69.7% of all next-of-kin contacted agreed to brain donation. While Caucasian families consented to donate brain tissue at a significantly higher rate (74.1%) than African-American families (57.0%) (*p*<0.001), African-American brain donation rates were as high as 60.5% in referrals from Maryland. Neither African-American nor Caucasian donors differed significantly from non-donors on any demographic or clinical factors ascertained, including age, sex, diagnosis of the donor, or in the relationship of the next-of-kin being contacted (*p*>0.05). However, Caucasian donors were significantly older, had more years of education, were more likely to be referred for study due to a psychiatric diagnosis, more likely to have comorbid substance abuse, and more likely to have died via suicide, as compared with African-American donors (*p*<0.05). When African-American participants are identified and approached, African-American families as well as Caucasian families are indeed willing to donate brain tissue on the spot for neuropsychiatric research, which supports the belief that African-American attitudes towards biomedical research may be more favorable than previously thought.

## Introduction

It is well-documented that African-American participants have been significantly under-represented in biomedical research participation, from areas such as clinical trials [1], organ donation (both antemortem and postmortem) [2–4], or for consent to donate biospecimens [5].

The lack of adequate research on African-American participants has the potential to impede the treatment and prevention of diseases such as neurological and neuropsychiatric diseases, since research findings on these illnesses may not necessarily be generalizable from Caucasian samples, and in the case of organ donation, race-mismatched donors can influence survival rates [6].

Several recent initiatives suggest that not only is the demand for donation of tissue specimen at the forefront in biomedical research, but the need for postmortem human brain donors with neuropsychiatric disorders as well as controls that includes minorities is imperative to advances in the field. The NIH Genotype-Tissue Expression (GTEx) Project [7] includes postmortem non-diseased healthy control specimens from multiple tissues of African-American and Caucasian donors as well as other minorities to understand human genetic variation. The CommonMind Consortium has examined schizophrenia and control prefrontal cortex in attempt to create a resource for studying genetic variants in this illness [8], as well as the BrainSeq Consortium which is studying RNA sequencing in control and neuropsychiatric brain tissue [9].

Postmortem human brain specimens from individuals with neuropsychiatric disorders such as schizophrenia, bipolar disorder, major depression, and post-traumatic stress disorder, as well as control specimens are critical to elucidate the genetic neuropathology of these illnesses, including the study of gene expression, DNA methylation, and proteomics as noted above [10–12]. Due to the heterogeneity and complexity of these disorders, large samples from diverse populations must be collected to uncover pathways for neuropsychiatric illnesses. Our research team has previously demonstrated the efficacy of studying large postmortem human brain datasets from both Caucasian and African-American samples for the study of transcription across the lifespan in human prefrontal cortex [13] and DNA methylation [14] but use of brain tissue for the study of neuropsychiatric disease is still described as somewhat underutilized [15].

Postmortem human brain donations are typically consented via one of two approaches. The first, prospective recruitment, whereby prospective donors are screened, give consent, and followed for a period of months or years until eventual brain removal at death, or the second, via medical examiner autopsy recruitment, in which protocols are designed such that referrals are provided at the time of autopsy via same-day, “cold-call” consenting from legal next-of-kin around the same time as organ donation. Both methods have relative advantages—with prospective recruitment allowing for a specific sample to be targeted and screened (e.g., Alzheimer’s disease patients participating in a given protocol), but do require patient tracking over a period of time, and tend towards more aged samples. In contrast, medical examiner recruitment samples require no long-term tracking, tend to be younger samples, and can yield relatively large numbers within a given window of time; however these samples come with potential confounds of drug abuse, suicide, and significant medical comorbidities [11].

Success in prospective recruitment of postmortem human brain donation in Caucasians has been previously documented, particularly in aged, upper middle class Caucasian samples, and most commonly in association with Alzheimer’s disease and neurodegenerative disease research, primarily in the form of registries for future “prospective” consent to brain donation [16]. The Netherlands Brain Bank has also demonstrated success with prospective brain donation registry recruitment in a Dutch sample, with over 1,000 psychiatric donors registered as of 2016 [17]. However, interpretation of success of prospective recruitment for brain donation tends to be measured on willingness or agreement to a future donation, rather than on actual brain accrual.

At least one study regarding success with on-the-spot, “cold-call”, medical examiner brain donations from next-of-kin in Caucasian samples has also been described by the New South Wales Tissue Resource Centre, where approximately 54% of families that were approached consented to brain donation [18] for psychiatric, substance abuse, and neurological disorders. In this same brain donation program of Caucasian brain donation samples, being future organ/transplant donors in other programs [19], or having prior conversations with loved ones about brain/organ donation before their death [20] were positively associated with brain donation.

Less is known about racial and ethnic minority participation in brain donation. The genetic risk architecture for neuropsychiatric diseases varies among racial groups, and while the genes may be the same, the pathological genetic variation within a single gene may vary by race. Insofar as African-American and sub-Saharan Africans may be under-represented in biomedical research, the age of personalized medicine demands that we explore all racial and ethnic groups in postmortem brain research in order to determine mechanisms of action specific to Black/African-American populations.

To our knowledge, just a handful of studies have assessed actual completion rates of brain donation among African-Americans, the majority of whom have focused on prospective neurodegenerative disease research [21–24]. Bonner and colleagues [25] improved rates of autopsy and brain donation consent in a study of strokes and dementia using a specialized recruitment program for African-Americans, which increased autopsy completion rates from 2% to 29%. In a longitudinal epidemiological study of a cohort of 784 African-Americans with Alzheimer’s disease who enrolled for study, 352 agreed to anatomical gifts (45% agreement); however, at the time of study, only 2.9% completed autopsy donations from this enrolled prospective sample at the time of publication [21]. Danner and colleagues found that in African-American control participants for an Alzheimer’s disease study, about 32% of participants agreed to yearly testing with eventual future brain donation, the majority of whom were female, and at the time of study only two deaths and one autopsy had resulted [23]. The autism brain banking consortium, while not providing data on rates of consent in given racial and ethnic minority groups, reported that 13.8% out of 94 autism-spectrum disorder brain donors were African-American, but no other information was included as to screening and referral data [24]. Although these studies demonstrate success in brain donation in African-Americans, prospective studies typically report willingness for future donation, and cannot be directly compared to actual brain accrual rates in a given population. Furthermore, the majority of studies with actual completion rates have focused on neurodegenerative disease in both Caucasian and African-American samples.

Very little is known about accrual of postmortem human brain donations for neuropsychiatric research in African-Americans. In fact, no study to our knowledge has specifically examined rates of brain donation and factors associated with consent to donate brain tissue for neuropsychiatric studies in African-Americans as compared with Caucasian participants, via “cold-calls” on the day of autopsy using a medical examiner sample.

Given the historical context and previous research on African-American research participation, coupled with our own experience with consenting African-American brain donors, we hypothesized that African-American participation in brain donation would be significantly lower than in Caucasian families. We conducted a retrospective review of consecutive referrals for postmortem human brain donation to determine which demographic and clinical characteristics might be associated with increased or decreased participation among African-American families.

## Methods

### Participants

All referrals for potential brain donation (*N* = 1,421) were individuals being autopsied at one of the three area medical examiners in the DC/Baltimore metropolitan area [e.g., (1) Washington DC (DC); (2) Virginia, Northern District (VA); and (3) Baltimore, Maryland (MD) sites), and were identified during daily morning rounds as either having a history of psychiatric symptoms or as a possible healthy non-psychiatric control. For all three sites, only African-American and Caucasian referrals were included in this study (due to very small numbers of Asian and Hispanic individuals being referred for autopsy, which would not allow for adequate power for statistical analysis).

First, referrals for possible brain donation (*n* = 266) for the National Institute of Mental Health (NIMH) Human Brain Collection Core (formerly in the Section on Neuropathology of the Clinical Brain Disorders Branch), NIMH Intramural Research Program, NIH, were identified through two local area medical examiners: the Office of the Chief Medical Examiner of the District of Columbia (OCME-DC), and the Office of the Chief Medical Examiner of Virginia (OCME-VA), Northern District, in Manassas, VA, according to NIH protocol #90-M-0142. Secondly, referrals for possible brain donation (*n* = 1,155) for the Lieber Institute for Brain Development (LIBD) Brain Tissue Collection in Baltimore, MD were identified through the Office of the Chief Medical Examiner for the State of Maryland (OCME-MD) between 2012–2015, according to the Maryland Department of Health and Mental Hygiene (MD-DHMH) protocol #12–24.

### Procedure and data collection

For all three sites, on the morning of referral from the medical examiner, either a board-certified neurologist (TMH) or a board-certified psychiatrist (JEK) contacted the next-of-kin to describe the study using an IRB-approved telephone script, and to obtain informed consent from the donor’s surviving legal next-of-kin, according to NIH protocol #90-M-0142 or MD-DHMH protocol #12–24. The two principal investigators with over 25 years (TMH) and 35 years (JEK) of experience, respectively, conducted telephone calls to next-of-kin for informed consent of tissue donation, with the first principal investigator (TMH) conducting the majority (~95%) of calls for this study. Both principal investigators (TMH and JEK) maintained a donation tracking log to collect all available demographic and clinical data on every referral to the brain donation program. This log included basic demographic data provided by each medical examiner, to include the age, race, sex, and presenting diagnosis or preliminary manner of death of the potential donor, as well as names of the decedent, next-of-kin, the relationship of the next-of-kin to the decedent, and contact information. When next-of-kin consented to donation, informed consent was audiotaped, and a 36-item telephone screening, i.e., the Lieber Institute for Brain Development Autopsy Questionnaire (see S1 Appendix), was completed to gather additional demographic, clinical, psychiatric history, substance abuse history, treatment, medical, and social history for all brain donors (please see S1 Appendix). When next-of-kin declined donation, contact was concluded with a family member, and no additional data was gathered on non-donors.

### Statistics

Data analysis was performed using R, version 3.5.1 (https://cran.r-project.org/). Categorical variables were analyzed using Pearson’s Chi-squared tests with Yates’ continuity correction, and continuous variables were analyzed using two sample unpaired t-tests with unequal variances. We used an alpha level of 0.05 for all statistical tests.

## Results

Among all three medical examiner referral sites, 1,213 out of 1,421 or 85.4% of referrals were reached, which included 239 NIMH next-of-kin of referrals reached (*n* = 100 from DC, *n* = 139 from VA), and 974 Maryland next-of-kin of referrals reached (see Table 1). Thus, the remaining 27 NIMH referrals (*n* = 6 from DC, *n* = 21 from VA) and remaining 181 Maryland referrals either did not respond to phone contacts, legal next-of-kin could not be reached, and/or did not have working phone numbers, and were considered non-responding referrals, with no further data collection possible.

**Table 1:** Postmortem human brain donation referrals and cases consented by medical examiner and race.

	Total Referrals	Families Reached	Yes to Donation	African-American Yes	Caucasian Yes
Site	*n*	*n* (%)	*n* (%)	*n* (%)	*n* (%)
Maryland	1,155	974 (84.3%)	707 (72.6%)	130 (60.5%)	577 (76.0%)
Virginia	160	139 (86.9%)	85 (61.2%)	7 (50.0%)	78 (62.4%)
DC	106	100 (78.7%)	54 (54.0%)	38 (48.7%)	16 (72.7%)
All Cases	1,421	1,213 (85.4%)	846 (69.7%)	175 (57.0%)	671 (74.1%)

Overall, the majority (*n* = 846, 69.7%) of 1,213 next-of-kin contacted at the time of their loved one’s death consented to postmortem human brain donation, including 57.0% of all African-American referrals that were contacted across the three offices (*n* = 175/307, see Table 1). Caucasian families consented to donate brain tissue at a significantly higher rate than African-American families [74.1% vs. 57.0%; OR = 2.15, *χ*^*2*^ (1, *n* = 1,213) = 30.8, *p*<0.001]. At the Maryland site, African-Americans donated at a rate of 60.5% (see Table 1).

### African-American donors vs. non-donors

African-American brain donors did not differ significantly compared to those that declined donation, e.g., non-donors. Specifically, there were no significant differences for age at death of donors (*M =* 45.6, *SD* = 17.4) as compared with non-donors (*M =* 44.7, *SD =* 18.0, *t*(269) = 0.42, *p* = 0.67). There were also no significant differences in the sex of the donors [% male: donors: 55.8% vs. non-donors: 59.1%, OR = 0.87, *χ*^*2*^ (1, *n* = 307) = 0.18, *p* = 0.67]. There were also no significant differences in African-Americans with respect to the kinship of the next-of-kin to the donor [rates of donation by parent, sibling, spouse, and child were: 60.6%, 57.9%, 56.5%, 54.2%, respectively, *χ*^*2*^ (3, *n* = 282) = 0.68, *p* = 0.88], nor in the sex of the next-of-kin being contacted for consent [% male: donors: 62.2% vs. non-donors: 55.0%, OR = 1.35, *χ*^*2*^ (1, *n* = 307) = 1.0, *p* = 0.32].

### Caucasian donors vs. non-donors

Caucasian donors and non-donors similarly had no significant differences in demographic data, including no significant differences for age at death (*M = 50*.*3*, *SD* = 17.9) as compared with declined referrals (*M = 50*.*7*, *SD =* 18.0, *t*(404) = 0.35, *p* = 0.73), or the sex of the donors [% male: donors: 75.1% vs. non-donors: 72.2%, OR = 1.16, *χ*^*2*^ (1, *n* = 906) = 0.76, *p* = 0.38]. There were also no significant differences in Caucasians with respect to the kinship of the next-of-kin to the donor [rates of donation by parent, sibling, spouse, and child were: 75.6%, 72.0%, 72.4%, 76.0%, respectively, *χ*^*2*^ (3, *n* = 864) = 1.40, *p* = 0.70], nor in the sex of the next-of-kin being contacted for consent [% male donors: 73.7% vs. 73.2%, OR = 1.03, *χ*^*2*^ (1, *n* = 870) = 0.01, *p* = 0.93].

### Reached families vs. non-responders

Although data was limited on non-responders (*n* = 208 or 14.6% of all referrals), we performed a series of sensitivity analyses to assess the generalizability of our findings. First, we confirmed that there were no demographic differences between families reached vs. non-responding referrals. Non-responding referrals did not differ from the responding donors above (*n* = 1,213) on race [*χ*^*2*^ (1, 1,421) = 0.07, *p* = 0.79], age [*t*(277) = -0.61, *p* = 0.54] or sex [*χ*^*2*^ (1, 1,421) = 0.12, *p* = 0.72]. African-American non-responding referrals (*n* = 55) were not significantly different from Caucasian non-responding referrals (*n* = 153) on any of the limited demographic data available for this subgroup, including age of donor at death [*M =* 46.5, *SD = 19*.*2* in AA, *M =* 48.9, *SD = 18*.*6* in Caucasian, *t*(96) = -0.77, *p* = 0.44], donor sex (60% Male in AA, 64% Male in Caucasian, [*χ*^*2*^ (1, *n* = 208) = 0.14, *p* = 0.71], although there was a trend towards a difference on history of psychiatric diagnosis in non-responding referrals between the two races [40.1% cases in AA, 57.0% cases in Caucasians, [*χ*^*2*^ (1, *n* = 208) = 3.54, *p* = 0.06].

### African-American vs. Caucasian donors

We then further examined detailed clinical data from donors to better untangle potential differences by race, for which full clinical characterizations were available on 806 donors (of 846 above, or 95.3%), to include 164 African-American and 642 Caucasian donors. We found that Caucasian donors were significantly older [*M =* 50.8, *SD = 18*.*2*, vs *M =* 45.9, *SD = 17*.*6* years, *t(250)* = 3.14, *p* = 0.002], had more years of education [*M =* 13.1, *SD = 2*.*6* versus *M =* 12.4, *SD = 2*.*3* years, *t*(258) = 3.26, *p* = 0.001], were more likely to be married [33.8% versus 22.6%, *χ*^*2*^ (4) = 18.8, *p* = 0.0008], were more likely to have died by suicide [18.5% versus 9.8%, *χ*^*2*^(1) = 6.6, *p* = 0.01], were more likely to have a psychiatric diagnosis [84.6% versus 75.4%, *χ*^*2*^(1) = 6.94, *p* = 0.008], and were more likely to have a comorbid substance use disorder [51.6% versus 42.1%, *χ*^*2*^(2) = 4.33, *p* = 0.04] (Please see Table 2). There were no differences in sex [65.6% versus 61.6%, *χ*^*2*^(1) = 0.75, *p* = 0.388], having a drug-related death [33.6% versus 26.8%, *χ*^*2*^(1) = 2.47, *p* = 0.11], or being a smoker [48.8% versus 54.4%, *χ*^*2*^(1) = 1.4, *p* = 0.23].

**Table 2:** Postmortem human brain donor characteristics.

	African-American (*n = 164)*	Caucasian (*n = 642)*	*p-*value
	Mean (SD) or *n* (%)	Mean (SD) or *n* (%)	
Age at death	45.9 (17.6)	50.8 (18.2)	<0.01**
Donor sex (% male)	101 (61.6%)	421 (65.6%)	0.39
Years of education	12.4 (2.3)	13.1 (2.6)	<0.001***
Married at time of death	37 (22.6%)	217 (33.8%)	<0.001***
Suicide death	16 (9.8%)	119 (18.5%)	<0.05*
Drug-related cause of death	44 (26.8%)	216 (33.6%)	0.11
History of psychiatric diagnosis	123 (75.0%)	543 (84.6%)	<0.01**
Comorbid substance use disorder	69 (42.1%)	331 (51.6%)	<0.05*
History of cigarette smoking	89 (54.4%)	313 (48.8%)	0.23

## Discussion

Over half (57.0%) of all African-American families, and over two-thirds (74.1%) of all Caucasian families who were contacted at the time of autopsy for consent to donate their deceased loved one’s brain for neuropsychiatric research agreed to do so. Thus, 69.7% of all families, regardless of race, consented to brain donation. Same-day, cold-calling referrals through medical examiner recruitment of postmortem human brain donation at the time of death in the Baltimore/DC/Northern Virginia metropolitan area over approximately a five year period yielded donations from 175 African-American donors, which may be the largest number of African-American brain donations reported to date in the peer-reviewed literature for neuropsychiatric research. This finding adds some validity to the growing body of literature suggesting that African-Americans are indeed amenable to participate in research, and our findings are the first to document this pattern in the research area of medical examiner brain donation for neuropsychiatric research outside of prospective Alzheimer’s disease research programs and registries.

None of the basic demographic variables including age at death, sex of donor, sex of next-of-kin family member, or the relationship of the next-of-kin to donor were significantly associated with African-American brain donation as compared with non-donors, as was also the case between Caucasian donors and non-donors. Although a previous study has indicated that non-working contact information has been an impediment to African-American research recruitment [26], we found comparable rates of non-responsiveness between both African-American and Caucasian referrals (both ~17%), as well as no significant differences between reached as compared to non-responding referrals.

We did, however, find several interesting differences between African-American and Caucasian brain donors. First, our Caucasian donors were significantly older than our African-American donors by nearly 5 years. It was not immediately clear from the available data why this was true; particularly because our Caucasian donors had significantly more deaths by suicide (18.5%) as compared with African-American donors (9.8%), which does not support the finding of Caucasian donors being older. This could somehow be related to who is referred for autopsy within this metropolitan area, or related to medical comorbidities of those being autopsied, but these are speculation, and further exploration of this finding may be warranted in a future study. Of note, several previous research studies of organ donation and willingness at future brain donation have found that age was indeed positively associated with likelihood to donate [27, 28].

Secondly, although African-American donors had an average of one year less of education than Caucasian donors, our African-American sample had an average education level slightly above a high school degree, which could be linked to relatively high participation levels in this study. Several studies have previously found a positive association between education level and research participation in African Americans [22, 29], education level and African-American organ donation [30], education and mental health treatment [31], or even community education about clinical research and subsequent participation, suggesting that examining education levels or querying knowledge of informed consent and clinical research may also be especially useful data to consider in future studies. Furthermore, as previous studies have suggested, community involvement and education [32, 33], including that of religious entities and staff in the recruitment process [34], and previous exposure/participation in medical research [5, 35] are additional factors to be considered in the “education” of research participants. Although education level of declined referrals was not available for this study, and education of the next-of-kin being contacted was not ascertained, education is certainly an area of further exploration in association with both Caucasian and African-American brain donation for neuropsychiatric research going forward.

We also found that our Caucasian donors were significantly more likely to be married than our African-American donors. We were unable to find literature supporting the association between marital status and organ donation or prospective brain donation in the context of race, but we did find one report of prospective brain donation sample in Australia having marital rates of 49.2%, which indicates that this may be a possible population to target in the future when recruiting for brain donation [36], particularly in prospective studies when this factor can be considered ahead of time.

Finally, we found that our Caucasian donors were more likely to have died via suicide, to have a history of psychiatric illness, and to have a comorbid substance use disorder than our African-American donors. Given that our target populations of study include psychiatric illness and substance abuse, many of whom are referred to the morgue due to suicide, it is unsurprising to find these clinical histories within donors, but it is unclear why they were more prominent among Caucasian donors as compared with African-American donors. These three factors are possibly interrelated, as individuals or family members with previous experience with a given disease of study, have been reported in the literature to be more inclined to participate in biomedical research overall [22]. Therefore, experience with illnesses such as depression and drug abuse may have contributed to increased participation among Caucasian donors.

It is also possible that donors with psychiatric symptoms and their families may have been more likely to donate because they may have 1) gained a degree of trust with medical professionals while seeking professional help for their deceased loved one; 2) may have previously participated in other medical or psychiatric research; or 3) may have most likely been motivated by altruism, i.e., the desire to contribute to science if the donation of their deceased loved one’s brain would help alleviate human suffering [18].

While not specifically studied here, the nature and timing of professional contact with participants have also been associated with increased research participation in African-Americans on a whole [37]. In the case of same-day, “cold-calling” for donations at the time of autopsy, flexibility in timing is severely limited due to when autopsies are being performed. Therefore, the nature with which professional contact is handled with the next-of-kin may be the only thing within control for medical examiner recruitment. The very nature of brain donation requests require our investigators to demonstrate humility, honesty, integrity, and other human traits that transcend socially constructed barriers based on race and ethnicity. Our research investigators’ decades-long clinical training and experience in brain donation consenting have possibly demonstrated a familiarity, understanding, and mastery of the clinical and social skills needed to interface with these families in the midst of an emotionally devastating event.

Another final area of consideration not formally measured in our study was racial concordance between research investigators and donors. Our informed consent protocol script was identical for all participants, and was not tailored to any specific racial group, and was delivered by two Caucasian physicians/research investigators with multiple decades of clinical experience in conducting next-of-kin informed consent calls. It has been reported that when recruitment is deemed ethnically sensitive (i.e., taking care to explain the study in a detailed and clear fashion, to answer questions, to address potential religious concerns, etc.), African-American participation may also increase [25, 38].

Additionally, success of research recruitment, even with the lack of racial concordance between these two physicians and the African-American families they contacted, has been previously demonstrated by Fryer and colleagues [39], suggesting that although African-American research staff may be preferable as well as advantageous for African-American research recruitment, they were not essential for the engagement of African-American families in this particular study.

Our study has a few potential limitations to consider. First, we were limited in our ability to gather additional demographic and clinical variables on families who declined donation, which would have allowed for more detailed analyses of why potential donors may have opted out of donation, including demographic variables like socioeconomic status, religious beliefs, degree of trust/mistrust in the medical community, or education. Having these data might offer more insight into further increasing donations, but is logistically impossible when families decline donation. Second, when a referral is consented or declined, we do not formally ascertain reasons why as part of our telephone screening process (e.g., altruistic, religious, decedent’s wishes), which could also provide insight into why our donation rate, particularly in African-American families, is relatively high. Finally, we were unable to analyze potential factors associated with brain donation in our Asian-American families and those of Hispanic ethnicity within our brain donation program due to very small numbers of referrals from these groups, which could be an area of further exploration. While African-American families may have been historically underrepresented in clinical research studies, the rate of consent for brain donation in African-American families was higher than predicted and to our knowledge, has never been previously reported elsewhere in a “cold-called”, neuropsychiatric sample, suggesting that attitudes about research participation may have evolved. When referrals for brain donation are identified, approached with clinical sensitivity by expertly trained postmortem research staff, high success rates for brain tissue donation are attainable, even in African-American samples. Continued efforts to increase brain donation in African-American and other minority groups will allow for more targeted psychiatric genetic studies within and across racial groups, which may in turn lead to improved understanding and treatment of complex psychiatric disorders in minority populations.

## Supporting information

S1 Appendix LIBD autopsy questionnaire. Telephone screening form completed with next-of-kin for every brain donor.
(PDF)
