# Supplementary material for: African-American and Caucasian participation in postmortem human brain donation for neuropsychiatric research
Source: PLoS One. 2019 Oct 23;14(10):e0222565. doi: 10.1371/journal.pone.0222565 (PMC6808324; doi:10.1371/journal.pone.0222565)
Supplement: S1 Appendix — Telephone screening form completed with next-of-kin for every brain donor. (PDF) [file pone.0222565.s001.pdf]

Date:________________________ Study Flyer: ☐ Yes ☐ No

S1 Appendix: LIBD AUTOPSY QUESTIONNAIRE
Neuropathology Section

Interviewer: ☐ __________________

Decedent's Name: ____________________________________ DOB: __________________

Handedness: R L A

Place of birth: __________________

Ethnicity: ______________________

Next of Kin Name: ____

NOK Home Phone: __________________________ NOK Cell Phone: ____________________

NOK Address: ____

____

Relationship to Decedent: ☐ Mother ☐ Father ☐ Spouse ☐ Sibling ☐ Son ☐ Daughter ☐ Other: ________________

1. History of psychiatric or psychological care? ☐ No ☐ Yes
(If yes, specify age of onset, symptoms, frequency and type of therapy, and the use of any medications)

____

____

____

2. History of taking any psychiatric medication? ☐ No ☐ Yes

____

____

3. History of taking any prescribed medication for medical problems? ☐ No ☐ Yes
(If yes to either, list last medications and dose/frequency, list past use of antipsychotics, Lithium, SSRIs, etc.)

____

____

4. History of psychiatric hospitalization(s)? ☐ No ☐ Yes
(If yes, specify where, when, duration of hospitalization, and reason for hospitalization)

____

____

5. If decedent had history of depressed mood, it lasted most of the day, nearly every day x 2 weeks or more:

☐ Yes ☐ No ☐ Uncertain Duration of episode: ___________________________

Episode was: ☐ current at death ☐ past

Symptoms:
☐ depressed mood most of day (or tearful/ irritable)
☐ anhedonia (observed or reported)
☐ significant wt loss or gain or appetite changes
☐ insomnia or hypersomnia nearly every day
☐ psychomotor agitation or retardation nearly every day (observed by others)
☐ fatigue or loss of energy nearly every day
☐ feelings of worthlessness or excessive/inappropriate guilt nearly every day
☐ decreased concentration or inability to make decisions nearly every day

6. History of: ☐ recurrent suicidal ideation (w/ or w/o plan or attempts)

Comments: ____

____

7. History of suicide attempts? ☐ No ☐ Yes
(If yes, specify number, type, and any sequelae such as seizures, coma, etc.)

____

____

8. History of manic episodes (irritable, hyper, agitated, elevated, euphoric)? ☐ No ☐ Yes
(If yes, specify initial age of onset and age of most recent episode): ______________________

☐ Lasting at least one week OR ☐ lasting at least 4 days, ☐ mania occurred when drug/alcohol-free? If not, substance being abused when manic: ______________________________

characterized by:

Symptoms:
☐ decreased need for sleep
☐ psychomotor agitation/ increased goal-directed activities
☐ spending sprees
☐ excessive involvement in pleasurable activities
☐ pressured speech or more talkative than usual
☐ racing thoughts or flight of ideas
☐ distractibility/ inability to stay on one track

9. History of any psychosis? ☐ hallucinations ☐ paranoia ☐ other delusions

Comments: ____

____

10. History of any: ☐ anorexia ☐ bulimia ☐ other ED ☐ panic attacks ☐ generalized anxiety ☐ OCD
(If yes, please describe age of onset, symptoms, treatment):

____

____

11. History of alcohol abuse? ☐ No ☐ Yes
(If yes, specify number of years, amount of daily intake, history of DTs, liver disease, blackouts, tolerance, or legal, social, or occupational impairment)

____

12. History of substance abuse? ☐ No ☐ Yes
(If yes, specify type of drug, age of onset, frequency of use, last use, and history of overdoses)

____

13. History of detoxification? ☐ No ☐ Yes
(If yes, specify where and when, use of AA/NA)

____

If decedent has a possible history of Dementia, Parkinson's Disease, or is over the age of 60, ask 14-18; if not, skip ahead to 19.

14. History of memory loss? ☐ No ☐ Yes
   a. Short Term Memory specifically affected: ☐ No ☐ Yes
(If yes, specify beginning of issue and severity)

____

____

   b. Trouble remembering faces or familiar names: ☐ No ☐ Yes
   c. History of difficulty navigating indoors: ☐ No ☐ Yes
   d. Difficulty finding words or phrases to express themselves: ☐ No ☐ Yes
   e. Difficulty grasping situations or explanations (confusion): ☐ No ☐ Yes
   f. Did decent continue driving? ☐ No ☐ Yes
      (If no, specify when driving ceased, and reason why)
   g. Did decedent have difficulty navigating local streets: ☐ No ☐ Yes

____

15. Was decedent seeing a doctor or receiving medication for memory problems? ☐ No ☐ Yes
(If yes, specify which doctor, what medication, and duration of treatment)

____

16. Did decedent have a history of a caregiver, assisted living, or a nursing home? ☐ No ☐ Yes
(Specify location and duration of care)

____

17. History of incontinence (bladder and/or bowel control)? ☐ No ☐ Yes

____

18. History of motor abnormalities? ☐ No ☐ Yes

a. History of tremors (resting or postural): ☐ No ☐ Yes

b. History of gait abnormalities (shuffling, small steps, decreased armswing): ☐ No ☐ Yes

c. Vocal changes (soft or raspy voice, slurred speech, drooling): ☐ No ☐ Yes

d. Balance Problems: ☐ No ☐ Yes

____

19. History of seizures? ☐ No ☐ Yes

(If yes, specify seizure type and frequency, and any medications used to treat them)

Seizure Type_____________ Frequency______________ Medication__________________________

20. History of strokes? ☐ No ☐ Yes

(If yes, specify symptoms, residual deficits, and site of hospitalization)

____

21. History of engagement in sports with a high risk of repetitive brain injury, including boxing, football, mixed martial arts, lacrosse, rugby, or auto racing? ☐ No ☐ Yes

____

22. History of hypertension? ☐ No ☐ Yes

(If yes, specify age of onset and duration of treatment)

Age Onset _______ Treatment Details____

23. History of diabetes? ☐ No ☐ Yes

(If yes, specify age of onset and type of treatment, e.g. insulin vs. oral hypoglycemic agents)

Age Onset _______ Treatment Details____

24. History of migraines? ☐ No ☐ Yes

(If yes, specify age of onset and type of treatment, e.g. triptans, narcotic agents)

Age Onset _______ Treatment Details____

25. History of head trauma? ☐ No ☐ Yes

(If yes, specify age of injury and type of treatment)

____

26. History of smoking? ☐ No ☐ Yes

(If yes, specify age of onset, amount smoked in pack-years, and when the individual stopped smoking)

Age Onset _______ Duration _______ Age Offset _______ Last known packs per day _______

27. History of special education/LD? ☐ No ☐ Yes

(If yes, specify any special placement)

____

28. Level of education? (Specify highest grade completed)

____

29. Most recent employment? (Specify type of job and duration of employment in that position)

____

30. Did the decedent ever suffer from physical abuse, sexual abuse, or other abuse? ☐ No ☐ Yes

____

31. Was the decedent a veteran? ☐ No ☐ Yes

Branch ________________ Duration of Service ________________ Combat Seen ☐ No ☐ Yes

32. Did the decedent ever suffer from PTSD symptoms due to combat-related trauma or physical/sexual abuseor other type of trauma? ☐ No ☐ Yes

(If yes, note history of flashbacks, nightmares, intrusive memories, avoidance, or hypervigilance)

____

____

33. Marital Status: ☐ Single ☐ Married ☐ Divorced ☐ Separated ☐ Widowed

34. Biological children? _______

35. Family history of mental illness or substance abuse?

____

36. Family history of suicide? ☐ No ☐ Yes

____

☐ Informed that may be contact in several months for additional info by phone; and/or medical record releases may be sent.
